# Effect of Hypercholesterolemia, Systemic Arterial Hypertension and Diabetes Mellitus on Peripapillary and Macular Vessel Density on Superficial Vascular Plexus in Glaucoma

**DOI:** 10.3390/jcm12052071

**Published:** 2023-03-06

**Authors:** María Sanz Gomez, Ni Zeng, Gloria Estefania Catagna Catagna, Paula Arribas-Pardo, Julian Garcia-Feijoo, Carmen Mendez-Hernandez

**Affiliations:** 1 Department of Inmunology Ophthalmology and ORL, IIORC, Complutense University of Madrid, 28232 Madrid, Spain; 2 Ophthalmology Department, Central Defense Hospital “Gomez Ulla”, 28047 Madrid, Spain; 3 Ophthalmology Department, Hospital Clínico San Carlos, Institute of Health Research (IdISSC), 28232 Madrid, Spain

**Keywords:** glaucoma, OCTA, OCT, peripapillary vessel density

## Abstract

Background/Aims: Vascular factors are involved in the development of glaucoma, including diseases such as hypercholesterolemia (HC), systemic arterial hypertension (SAH), and diabetes mellitus (DM). The aim of this study was to determine the effect of glaucoma disease on peripapillary vessel density (sPVD) and macular vessel density (sMVD) on the superficial vascular plexus, controlling differences on comorbidities such as SAH, DM and HC between glaucoma patients and normal subjects. Methods: In this prospective, unicenter, observational cross-sectional study, sPVD and sMVD were measured in 155 glaucoma patients and 162 normal subjects. Differences between normal subjects and glaucoma patients’ groups were analyzed. A linear regression model with 95% confidence and 80% statistical power was performed. Results: Parameters with greater effect on sPVD were glaucoma diagnosis, gender, pseudophakia and DM. Glaucoma patients had a sPVD 1.2% lower than healthy subjects (Beta slope 1.228; 95%CI 0.798–1.659, *p* < 0.0001). Women presented 1.19% more sPVD than men (Beta slope 1.190; 95%CI 0.750–1.631, *p* < 0.0001), and phakic patients presented 1.7% more sPVD than men (Beta slope 1.795; 95%CI 1.311–2.280, *p* < 0.0001). Furthermore, DM patients had 0.9% lower sPVD than non-diabetic patients (Beta slope 0.925; 95%CI 0.293–1.558, *p* = 0.004). SAH and HC did not affect most of the sPVD parameters. Patients with SAH and HC showed 1.5% lower sMVD in the outer circle than subjects without those comorbidities (Beta slope 1.513; 95%CI 0.216–2.858, *p* = 0.021 and 1.549; 95%CI 0.240–2.858, *p* = 0.022 respectively. Conclusions: Glaucoma diagnosis, previous cataract surgery, age and gender seem to have greater influence than the presence of SAH, DM and HC on sPVD and sMVD, particularly sPVD.

## 1. Introduction

Primary open-angle glaucoma (POAG) is a progressive optic neuropathy that leads to retinal ganglion cell loss and is the leading cause of irreversible blindness worldwide [1]. The physiopathology of glaucoma is not well known. Intraocular pressure (IOP) increase is the main risk factor for both the development and progression of glaucoma and the only one on which successful therapeutic action can be conducted [2], but there are other known risk factors [3,4,5,6]. Retinal microvasculature and vascular disfunction plays an important role in the development of glaucoma, and one of the leading proposed mechanisms of retinal ganglion cell injury is based on the vascular hypothesis [7,8]. The mechanisms of glaucomatous damage to the optic nerve head (ONH) remain controversial. The mechanical hypothesis, centered on the effect of high IOP on the retinal ganglion cell axons within the lamina cribosa, and the vascular hypothesis focused on vascular dysregulation and its association with ONH blood supply, can be closely related [9]. Multiple vascular factors can be involved, including diseases which are highly prevalent such as atherosclerosis, hypercholesterolemia (HC), systemic arterial hypertension (SAH), and diabetes mellitus (DM) [10,11,12,13].

The retina is considered as a window to the whole body. It is a unique site where the in vivo microvasculature can be directly visualized and monitored over time, which shares similar physiological features with vital organs such the brain, the heart, or the kidney [14,15,16]. Some studies have suggested that the retinal microvasculature could reflect the systemic circulation in vivo [17].

Retinal microvasculature visible in retinography can also provide information on the status of the systemic circulation in vivo [18]. On the other hand, optical coherence tomography angiography (OCTA) is a novel, non-contact, non-invasive imaging method that provides a three-dimensional assessment of ocular microcirculation. It generates data on the microvascular structure of the eye by measuring and processing the notion contrast of intravascular erythrocytes by sequential optical coherence tomography (OCT) scans of a particular area of the retina [19]. With this non-invasive tool, circumpapillary, papillary, and macular vessel density have been shown to be lower in glaucoma patients [20].

The main objective of this prospective, single center, observational study was to determine the effect of glaucoma disease on peripapillary vessel density (sPVD) and macular vessel density (sMVD) on superficial vascular plexus measured with OCTA controlling differences on comorbidities such as SAH, DM and HC between patients diagnosed with glaucoma, glaucoma suspects, and normal subjects.

A secondary objective of the study was to compare the diagnostic ability of OCTA in glaucoma patients.

## 2. Material and Methods

### 2.1. Study Design

This was a prospective, unicenter, observational cross-sectional study, approved by the Institutional Review Board of our university hospital, the Hospital Clínico San Carlos of Madrid, and carried out in accordance with the principles of the Declaration of Helsinki. Patients with glaucoma, glaucoma suspects, and healthy control subjects were prospectively enrolled. Written informed consent was obtained from each participant who fulfilled the eligibility requirements for inclusion in the study.

### 2.2. Study Participants

All glaucoma patients who attended glaucoma consultation for a routine exam, who presented minimal best-corrected visual acuity (BCVA) of 20/40, clinically determined glaucomatous optic disc changes, repeatable glaucomatous visual field loss, or both, and open-angle on gonioscopy were eligible for inclusion.

An abnormal visual field was defined as a mean defect (MD) greater than 2 dB or depressed points in the defect curve and variance loss higher than 7 dB^2^ using the Octopus TOP G1 program, Octopus 600, Haag Streit AG, Bern, Switzerland.

Glaucomatous optic disc changes were defined as focal (localized notching) or diffuse neuroretinal rim narrowing, or concentric enlargement of the optic disc cup.

Glaucoma suspects were considered those with a family history of glaucoma, or a former IOP increase either with or without hypotensive treatment.

Exclusion criteria were non-glaucomatous ocular disease, systemic disease or treatment affecting the visual field, refraction exceeding 5 D equivalent sphere or 3 D astigmatism, angle closure, or uveitic and neovascular glaucoma.

Control subjects were recruited with the following inclusion criteria: a minimal BCVA of 20/40, normal eye examination in both eyes, IOP < 20 mmHg, normal aspect of the optic disc, and no personal or familiar history of glaucoma.

### 2.3. Examination Protocol

For the assessment of the primary and secondary endpoints, once the informed consent had been signed, all participants were explored the same day upon the performing of a complete ophthalmologic examination, which included refraction and keratometry, BCVA, slit-lamp biomicroscopy examination, IOP measurement using the handheld Goldmann applanation tonometer (GAT) Perkins (Clement-Clarke, Haag-Streit, Harlow, United Kingdom) and the iCare 200 rebound tonometer (iCare, Tiolat Oy, Helsinki, Finland), pachymetry using the anterior segment module spectral domain optical coherence tomography (SD-OCT, Cirrus, HD-OCT 5000, software version 5.2; Carl Zeiss Meditec, Inc., 2020. AG, Jena, German) and funduscopy using a 90D lens.

Peripapillary retinal nerve fiber layer (RNFL) thickness, retinal ganglion cell complex analysis, average ganglion cell layer, inner plexiform layer (GCIPL), and superficial peripapillary and macular vessel parameters were automatically measured using the Cirrus HD-OCT 5000, software version 11.5.2.54532 (Carl Zeiss Meditec, Inc., 2020. AG, Jena, Germany), which automatically provides all parameters in the SD-OCT and OCT-A.

A visual field examination was conducted and analyzed for glaucoma patients, but not for all healthy control subjects, as per the protocol.

The independent variables analyzed were peripapillary vessel density and whole macular, outer and inner macular vessel density measured using OCTA.

The parameters of both SD-OCT and OCTA were independently, blindly, and prospective measured, since the values are automatically provided by the Cirrus, HD-OCT 5000 device.

Study variables such as demographics and medical/ophthalmological history were assessed by questionnaire and extracted from the patients’ medical file at the visit. The focus was on ophthalmological history (age-related macular degeneration (AMD), cataract, glaucoma, retinopathies, ocular surgeries and trauma), and history of SAH, HC and DM. In addition, in the clinical history, the reason for the prescription of medication and the medical background of every subject included in the study were checked.

Only one randomly selected eye per subject was included in the final statistical analysis.

### 2.4. Optical Coherence Tomography Angiography

The Cirrus HD-OCT 5000, software version 11.5.2.54532 (Carl Zeiss Meditec, Inc., 2020. AG, Jena, German) not only automatically calculates the peripapillary RNFL, the segmented ganglion cell complex, and papillary cup parameters, but also allows for the measuring of vessel density on the superficial vascular plexus in an area previously determined by the device. It has a 68-kHz axial scan repetition rate per second. This provides a transverse and axial resolution of 15 and 5 microns, respectively. The Cirrus 5000 software Angioplex^TM^ automictically quantifies the superficial plexus, which is defined from the inner limiting membrane to the inner plexiform layer. Blood flow information is generated by the algorithm OMAG (optical microangiopathy) with 245 A-scans in each B-scan.

All scans were performed by the same operators, who were blind to the diagnosis (MS, NZ, and EC), with pupil dilation in a dark room on the same day as the other tests. Poor-quality scans with a signal strength index (SSI) < 8 were excluded from the analysis.

**Figure 1:**
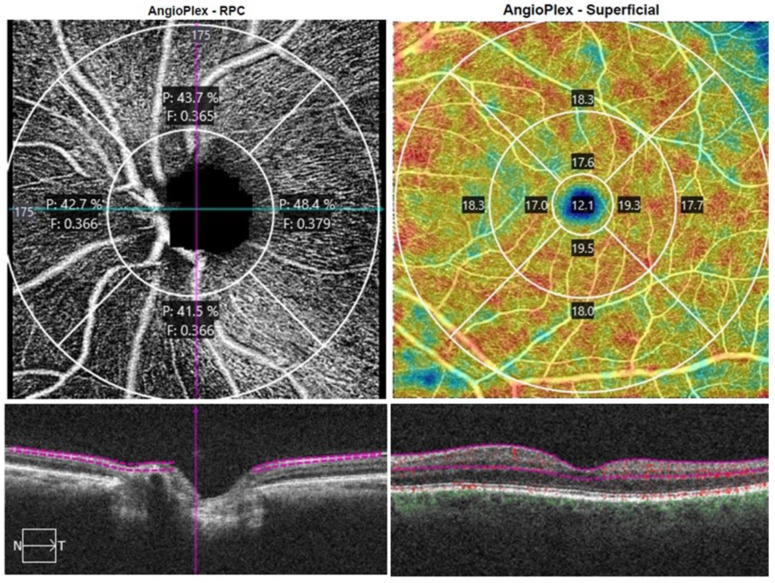
Peripapillary and macular vessel measurements offered by Angioplex OCTA in a glaucoma patient. Angiography slabs of the optic nerve head from a 4.5 × 4.5 mm scan showing the radial peripapillary capillary (**left**). Superficial angiography slabs of the 6 × 6 mm macular scan obtained using the OCT Cirrus 5000 (**right**).

Peripapillary 4.5 × 4.5 mm scans centered on the ONH were obtained. Two peripapillary parameters were measured in the area located between an inner circle with a radius of 2 mm and an outer circle with a 4.5 mm radius, both centered on the ONH, divided into four quadrants: superior, inferior, temporal, and nasal, the peripapillary perfusion density and the flux index for the whole peripapillary circle, and the four sectors. Perfusion density is the total area of perfused radial peripapillary capillary vasculature per unit area in a specific region (%), and flux index is a dimensionless parameter between 0 and 1 representing the average decorrelation signal (Figure 1).

Macular 6 × 6 mm scans centered on the fovea were performed. The OCTA Cirrus 5000 software subdivides the macular scan into the Early Treatment of Diabetic Retinopathy Study (ETDRS) map: a central circle within the 1-mm central circle on the ETDRS grid, an inner circle from 1 to 3 mm, an outer or external circle from 3 to 6 mm, and the whole circle.

Macular vessel density is defined as the total length of blood vessels from the skeletonized image to the total area in mm ratio and macular perfusion density, defined as the total area of perfused vasculature per unit area (%), and foveal avascular zone (FAZ) were automatically measured. The FAZ area, defined as the macular foveal area without blood flow signal (mm^2^), FAZ perimeter (mm), and the acicularity index (AI) i.e., the measured perimeter of FAZ-to-the perimeter of the projected circle ratio with the same area as the FAZ, was collected (Figure 1).

### 2.5. Statistical Analysis

The aim of this study was to evaluate the effect of suffering glaucoma on peripapillary and macular vessel density measured with OCTA controlling differences as to SAH, HC and DM between glaucoma diagnosed patients, glaucoma suspects, and normal subjects.

For this purpose, a linear regression model with 95% confidence and 80% statistical power was performed.

The Kolmogorov-Smirnov test was used to confirm the normal distribution of quantitative data.

A descriptive analysis of the variables included in the study was performed. The calculation of frequency distributions for qualitative variables and measures of central tendency and dispersion, mean, standard deviation [SD] in variables with normal distribution, and median and interquartile range in those variables with non-normal distribution for quantitative variables were performed. The 95% confidence intervals (95%CI) were shown for results associated with the primary and secondary objectives.

Sex, the percentage of patients who had undergone cataract surgery, patients with SAH, DM and HC, differences between the normal subjects and the glaucoma patients’ groups were compared using the Pearson χ^2^ test. The Student’s t-test was used to compare measurements between the two groups in parameters with normal distribution. Parameters not following normal distribution were evaluated with non-parametric tests. Relationships between parameters were analyzed using the Pearson and Spearman correlation coefficients.

Receiver operating characteristic (ROC) curves were constructed and areas under the ROC curves (AUROCs) were used to assess the capacity of each OCTA and SD-OCT variable to distinguish between glaucomatous and healthy eyes. AUROCs were compared for the different parameters using the DeLong method [21].

All statistical tests were performed using the software package IBM SPSS (version 26.0; IBM Corp., Armonk, NY, USA). Significance was set at *p* < 0.05.

## 3. Results

**Figure 2:**
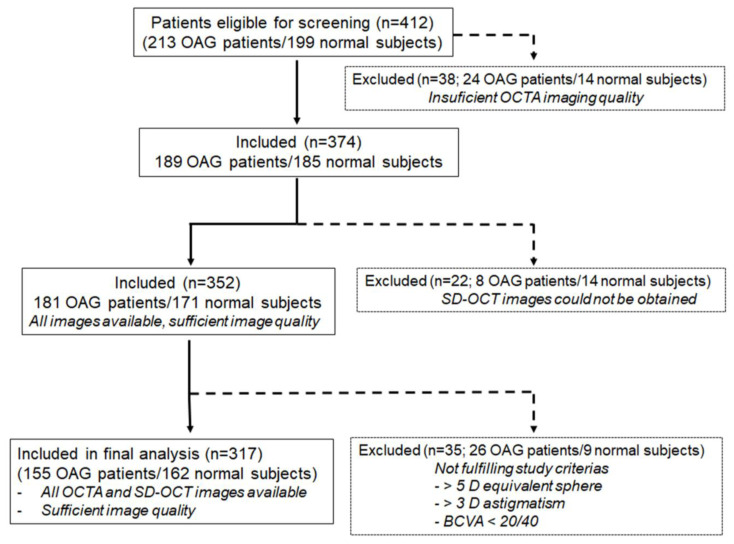
Flowchart of the patient enrollment process of the study.

A total of 412 subjects scheduled for ophthalmology consultation were eligible for screening: 213 glaucoma patients and 199 control subjects. Out of these 412, 38 did not have enough quality macular and ONH OCTA scans in at least one eye; therefore, images were not available for analysis. The primary reason for poor OCTA image quality was the high quota of motion artifact, making analysis unfeasible. Twenty-two subjects were excluded from the analysis because ONH or macular images with SD-OCT could not be obtained. Finally, a further 35 were excluded from the statistical analysis because the inclusion/exclusion criteria were not satisfied, mainly because of refraction exceeding the 5 D equivalent sphere or 3 D astigmatism and visual acuity <20/40. As a result, only 317 out of the 412 subjects that were initially eligible for screening were included in the final analysis. (Figure 2).

### 3.1. Analysis of Demographics

The data of 317 subjects were included in the final analysis: 155 glaucoma patients or glaucoma suspects, and 162 normal subjects, 55.8% of which male. The mean age was 58.27 years.

Eighty-eight subjects were pseudophakic (27.8%). A total of 38.8% of patients suffered from SAH, and 13.9% were diabetic, none of whom presented clinically significant diabetic macular edema or proliferative diabetic retinopathy, and 28.4% had a history of HC. There were significant differences in age, gender, patients having undergone cataract surgery, DM, HC, and SAH between glaucoma patients and control subjects. There were no differences in BCVA, pachymetry, and the disc area between groups. Detailed baseline demographics are presented in Table 1.

### 3.2. Qualitative Analysis

Out of 155 glaucoma patients, eighteen had undergone glaucoma surgery (11.6%), of which 15 had undergone surgery once and three presented refractory glaucoma, two of them undergoing reintervention once, and a single patient undergoing reintervention twice.

Fifty-two glaucoma patients (32.2%) were on antiglaucomatous treatment. Twenty-four (15.5%) were taking a single drug, twenty-five were taking (16.1%) two drugs, and three patients (1.9%) were under three antiglaucoma medications, while forty-three patients (27.7%) remained without ocular hypertensive treatment (Table 1).

### 3.3. Quantitative Analysis

IOP values measured with iCare 200 and GAT, RNFL, as well as ganglion cell complex results evaluated using SD-OCT are shown in Table 1. Statistical differences were found between healthy and glaucomatous subjects in all parameters evaluated with SD-OCT except disc area and GCIPL, which was similar in both groups, 1.84 vs. 1.85 mm^2^ and 264.88 ± 29.55 vs. 258.25 ± 30.49 µ in glaucoma and healthy subjects, respectively. The thinnest RNFL and greatest cup volume and cup-to-disc ratio were found in patients included in the glaucoma and glaucoma suspects group.

In Table 2, the results of the peripapillary and macular vessel density analysis performed with OCTA are shown.

Significant differences between glaucoma patients and normal subjects in whole peripapillary and macular vessel density were found (44.92 ± 1.65 vs. 43.22 ± 2.64%; <0.0001 and 43.72 ± 4.55 vs. 40.88 ± 5.75; *p* < 0.001). In the foveal area, however, AI showed significantly lower values in glaucoma subjects (0.68 ± 0.11) than in controls (0.71 ± 0.09), *p* < 0.005; both the FAZ area and the perimeter did not show differences between the groups, finding slightly inferior values in glaucoma patients in FAZ area, 0.21 ± 0.13 mm^2^ in glaucomatous subjects vs. 0.23 ± 0.11 mm^2^ in controls, *p* = 0.191, and similar values in FAZ perimeter, 1.96 ± 0.75 mm in glaucoma subjects vs. 1.96 ± 0.50 mm in control group, *p* = 0.929. Macular perfusion density in the central circle did not show differences between the groups; however, vessel density in this macular sector showed slightly lower values in glaucoma patients: 20.18 ± 8.35% vs. 21.38 ± 7.44%, *p* = 0.177. Significantly lower vessel density values were found in the two concentric macular external rings or circles in glaucomatous patients (Table 2).

Both peripapillary vessel density and flux index showed significatively lower values in glaucoma patients and glaucoma suspects, not only globally, but also in all peripapillary sectors analyzed with OCTA.

In Table 3, most relevant correlations between peripapillary and macular vessel density parameters analyzed with OCTA and demographic data, RNFL thickness, papillary and macular parameters obtained with SD-OCT are shown.

Significant negative moderate correlation between age and number of antiglaucomatous medications and peripapillary vessel density in all peripapillary sectors as well as most parameters that quantify macular vessel density were found, the greatest being the correlation between the number of antiglaucomatous medications and the peripapillary perfusion density in the inferior quadrant (Pearson’s correlation coefficient −0.443, *p* < 0.001).

Regarding the RNFL, ONH and ganglion cell complex parameters analyzed with SD-OCT, a positive correlation between RNFL thickness and peripapillary vessel density parameters was observed, with the greatest the correlation found between RNFL thickness and peripapillary perfusion density in the superior quadrant (Pearson’s correlation coefficient 0.429, *p* < 0.0001). Parameters related to size of the ONH cup, i.e., cup-to-disc ratio, vertical cup-to-disc ratio, and cup volume showed a low negative correlation with peripapillary vessel density; that is, the greatest cup-to-disc ratio and cup volume, and the least peripapillary vessel density. On the other hand, significant positive moderate correlation between peripapillary vessel density indices and rim area were found, the greatest being the correlation between rim area and peripapillary perfusion density in the inferior quadrant (Pearson’s correlation coefficient 0.502, *p* < 0.0001). The greater the rim area, the higher the peripapillary vessel density.

A significant but low negative correlation between ganglion cell complex measurement and most of peripapillary and macular vessel density parameters was found (Table 3).

Table 4 shows the results of the multiple regression model.

Whole peripapillary and macular vessel density, peripapillary and macular vessel density in each peripapillary and macular sector, foveal avascularity indices, FAZ area, FAZ perimeter, and AI were considered dependent variables. Independent variables included in the regression model were glaucoma diagnosis, SAH, DM and HC, and all demographic data that showed differences between both diagnostic groups, including age, gender, and previous cataract surgery.

Parameters with greater effect on peripapillary vessel density were glaucoma diagnosis, gender, pseudophakia, and DM. These parameters showed an effect on both whole peripapillary vessel density and vessel density in the superior and inferior peripapillary quadrants. Glaucoma patients had a peripapillary vessel density 1.2% lower than healthy subjects (β slope 1.228; 95%CI 0.798–1.659, *p* < 0.0001). Women presented 1.19% more peripapillary vessel density than men (β slope 1.190; 95%CI 0.750–1.631, *p* < 0.0001), and phakic patients presented 1.7% more peripapillary vessel density (β slope 1.795; 95%CI 1.311–2.280, *p* < 0.0001). Furthermore, DM patients had 0.9% lower vessel density than non-diabetic patients (β slope 0.925; 95%CI 0.293–1.558, *p* = 0.004). SAH and HC did not affect all the peripapillary vessel density analyzed except the peripapillary perfusion density in the nasal quadrant (β slope 1.205; 95%CI 0.389–2.020, *p* = 0.004).

With regard to the factors that can influence macular vessel density, their behavior was different. Glaucoma diagnosis does not seem the parameter with the greatest effect on macular vessel density. Again, phakic patients showed a macular vessel density higher than pseudophakic patients, 1.9% higher in the outer circle (β slope 1.921; 95%CI 0.568–3.274, *p* = 0.006), while patients with SAH and HC showed 1.5% lower macular vessel density in the outer circle than subjects without those comorbidities (β slope 1.513; 95%CI 0.216–2.858, *p* = 0.021 and 1.549; 95%CI 0.240–2.858, *p* = 0.022, respectively.

Given the significant age difference between normal subjects and glaucoma patients, to examine the effect of age in the analyses, a comparison of a reduced age-matched cohort of healthy subjects versus the glaucoma group was performed. The results are shown in Tables S1–S4 in Supplementary Materials. No relevant differences were found in the results of this sub-analysis and the results obtained in the analysis performed including all the subjects enrolled in the study.

Table 5 shows AUROCs for each OCTA and ONH SD-OCT parameter evaluated in this study and the cut-off providing sensitivity for 95% and 80% specificity determined in each case.

Overall, the AUROCs for discriminating between healthy and glaucomatous eyes were higher for the OCTA peripapillary blood flux index in the nasal quadrant, whole peripapillary blood flux index, and peripapillary perfusion density in the inferior quadrant: 0.79; 95%CI 0.73 to 0.85, *p* < 0.001 vs. 0.78; 95%CI 0.0.72 to 0.85, *p* < 0.001 vs. 0.78; 95%CI 0.72 to 0.84, *p* < 0.001. The OCTA macular vessel density indices and ONH SD-OCT parameters showed lower AUROCs. Macular vessel density in the outer circle and SD-OCT rim area showed the highest AUROCs for OCTA macular and SD-OCT indices: 0.75; 95%CI 0.68 to 0.81; *p* < 0.001 vs. 0.74; 95%CI 0.67 to 0.81, *p* < 0.001, respectively.

The SD-OCT rim area had the greatest sensitivity, with a specificity of 95% (Whole peripapillary blood flux index had a sensitivity of 29%, a positive likelihood ratio of 5.85, and a negative likelihood ratio of 0.74 and SD-OCT rim area had a sensitive of 44%, a positive likelihood ratio of 8.8 and a negative likelihood ratio of 0.58) and a specificity of 80% (whole peripapillary blood flux index had a sensitivity of 65%, a positive likelihood ratio of 3.26, and a negative likelihood ratio of 0.43, and the SD-OCT rim area had a sensitivity of 60%, a positive likelihood ratio of 3.02, and a negative likelihood ratio of 0.49).

Pairwise comparisons showed that the AUROC of the whole peripapillary blood flux index (0.78) was not significantly different than the SD-OCT rim area (0.74) (*p* = 0.628) or macular vessel density in the outer circle (0.75) (*p* = 0.332).

## 4. Discussion

Although the main risk factor in glaucoma development is IOP increase [22], vascular theory is included among theories of this pathology development. Vascular theory explains vascularization importance because it is responsible for the nutrient supply to the retinal ganglion cells. It is likely that both risk factors come together, having the greatest weight on the mechanical effect of IOP increase on the papilla [9].

OCTA quantitatively measures vascular density the in retina using an indirect method based on erythrocyte movement inside the blood vessels [19].

The clinical utility of OCTA has been demonstrated in different pathologies with retina vascular damage such as diabetic retinopathy [23]. It has been found that patients with SAH present macular vessel density reduction in their central area, and concrete foveal and parafoveal levels [24]. A recent study found microcirculatory disturbances in patients with familiar HC [25]. The prospective follow up of these patients with SAH, DM and HC using OCTA could help to identify vascular changes that might help with better metabolic and therapeutic disease control.

Despite the importance of vascular factors in glaucoma development, the role of vascular risk diseases such as SAH, DM or HC has not been significantly studied in glaucoma patients.

In this study, the effect of those diseases on superficial retinal vessel density has been analyzed, evaluating the effect of glaucoma on peripapillary and macular vessel density, controlling for differences in comorbidities such as SAH, DM or HC. Our results show that some clinical factors in patients have greater weight on macular and peripapillary density than the presence of SAH, DM and HC themselves. Phakic patients present higher macular and peripapillary density, its most marked effect being at some peripapillary quadrants, obtaining values 2.22% above in vessel density in some peripapillary sectors such as the peripapillary perfusion density in the inferior quadrant in phakic patients, which is in concordance with what has been formerly published [26].

Macular vessel density is influenced by age and gender, but peripapillary density is not. For each year-old increase, macular vessel density decreases 0.1% in the central and inner macular circle.

With respect to the effect of age on vascular density, a relationship with central macular vessel density has been found. Our study agrees with those previously performed on healthy subjects. Abay et al. evaluated macular and papillary and peripapillary vessel density on healthy subjects in different age groups, finding a decrease in total parafoveal and perifoveal macular density with no differences in the foveal avascular zone [27]. Changes in peripapillary vessel density found by other authors in different age groups are in concordance with our results, as we have not found an age effect on peripapillary vessel density [28,29]. On the contrary, macular vessel density was lower as age increased. For each year the age increases, macular vessel density decreases 0.081%, and is more accentuated in macular perfusion density in the inner circle and macular perfusion density in the central circle, which is in line with previous results [27]. The reason for these discrepancies might be the methods used for measuring vessel density. The software we used was different from those used in these studies.

With regard to vascular density differences determined with OCTA between gender, women present a 1.1% increase in peripapillary vessel density compared to men, this association being more evident in some peripapillary sectors such as the inferior and superior, and not finding a gender effect on macular vessel density. Comparative retinal vascular density studies examining gender are scarce. A former study performed by Wang et al. in 111 healthy subjects and 130 glaucomatous subjects found that women presented a 1.2% and 0.7% significant increase in circumpapillary vessel density and macular vessel density, respectively, compared to men [30]. This trend was greater in glaucomatous patients in an early stage. In our study, the mean defect of glaucoma patients was 2.5 dB, that is, there is a predominance of early glaucoma, which is in line with Wang et al.’s findings [30].

In this study, glaucoma diagnosis seems to have more effect on peripapillary vessel density than the presence of vascular risk factors such as SAH and HC. In our study, suffering DM supposes a peripapillary vessel density that is 0.9% lower. Suffering SAH or HC does not show an effect on peripapillary vessel density. However, having undergone cataract surgery, or being a woman, seems to have more relevance on peripapillary vessel density, but not greater than whether one suffers from glaucoma.

Except for this effect of DM on peripapillary vessel density, the influence of these three diseases on vascular density determined by OCTA has not been shown in our results. Furthermore, glaucoma diagnosis, the presence of pseudophakia, sex, and age seem to have more of an effect on the evaluated parameters than the fact of having high blood pressure, DM or HC.

With regard to the diagnostic capacity of the measurement of vessel density in glaucoma, when evaluating differences on macular vessel density between glaucoma patients or glaucoma suspects and healthy subjects, differences were found in whole macular perfusion density, macular perfusion density in the outer circle, and macular perfusion density in the inner circle, but macular vascular indices did not show statistically significant differences between both groups in macular perfusion density in the central circle, FAZ area (mm^2^), foveal avascular perimeter (mm), and AI. However, peripapillary vessel density did show differences between both groups, both globally and in sectors. AUROCs results also show a higher diagnostic performance in peripapillary vessel density than macular vessel density. Our results are in line with other studies that found that the diagnostic capacity of peripapillary vessel density showed higher diagnostic performance [31,32].

In addition, a correlation exists between peripapillary vessel density and RNFL thickness, so that patients with lower RNFL thickness, and therefore advanced structural damage, would present lower vascular density. This does not happen with retinal ganglion cell complex indices. Glaucoma studies performed by other authors have demonstrated diagnostic superiority on RNFL over retinal ganglion cell complex in patients with early glaucoma, ganglion cell complex analysis being considered to be useful for detecting glaucoma in myopic eyes or advanced glaucoma, in which structural features such as peripapillary atrophy or a potential floor effect may reduce the diagnostic ability of peripapillary RNFL analysis [33,34]. In our study, AUROCs are inferior by 0.8 and slightly superior in peripapillary vascular density indices. The fact that glaucoma patients, whose mean defect is 2.5 dB, have been selected and glaucoma suspects without ocular hypotensive treatment have been included in the glaucoma group, could explain those results.

Our study has several limitations. A visual field examination was done for glaucoma patients, but not for all healthy control subjects, so the analysis of mean deviation in glaucoma patients was not assessed.

Axial length could influence OCT imaging. In this study, axial length was not measured, but the refractometry was. Patients with a refraction exceeding a 5 D equivalent sphere or 3 D astigmatism were excluded, and therefore the effect of axial length on OCT imaging should be irrelevant.

Glaucoma patients and healthy subjects present differences in SAH, DM and HC distribution, as well as age, sex or pseudophakia differences. However, primary objectives such as the determination of the effect of suffering glaucoma on peripapillary and on macular vessel density measured with OCTA, controlling comorbidities such as SAH, DM and HC identification of patient ophthalmologic exam factors that influence AOCT parameters, were adjusted for those confusion factors. Thus, we believe that those differences did not affect the results.

On the other hand, the regression model used to evaluate the presence of glaucoma, clinical examination parameters, and SAH, DM and HC comorbidities reached sufficient statistical power, above 80% and 95% confidence levels.

All analyses performed on DM or HC have a lower statistical power. A great number of diabetic or HC patients would be needed to definite conclusions; however, due to our hospital casuistry this sample size cannot be reached. No matter its low statistical power, our results are relevant as a trend is detected.

Prior to the start of this study, a sample size estimation was performed by comparing the mean differences in OCTA whole peripapillary vessel density between groups.

To evaluate the effect of SAH, DM and DL on peripapillary vessel density, 65 SAH and no-SAH patients needed to be included: 34 HC and non-HC patients and 24 diabetic and non- diabetic patients.

Our research has focused on vessel density quantitative metrics derived from OCTA images, but skeletonized vessel density was not evaluated. Since skeletonization could narrow vessels to a width of a single pixel, it does not create a vascular density, but rather becomes a length-based measurement.

The Cirrus HD-OCT 5000 Angioplex has built-in software to automatically calculate vessel density. The results shown in this study have been derived from the quantitative data automatically provided by this device, and no additional quantification has been performed on the images obtained for each subject. The underlying algorithms used have not been made publicly available, and calculated OCTA quantitative metrics cannot be generalized beyond this device.

There are no studies that evaluate the relevance of these risk factors in glaucoma patients. More studies are needed to evaluate the effect on different glaucoma types and possible differences between primary open angle and normotensive glaucoma. Time since diagnosis, hemoglobin A1C value in diabetic patients, or blood pressure values should also be assessed in future studies.

In conclusion, glaucoma diagnosis has more of an effect on superficial peripapillary and macular vessel density than comorbidities such as SAH, DM and HC in glaucoma patients, glaucoma suspects and normal subjects. Other factors such as cataract surgery, age and gender seem to present a greater influence than the presence of SAH, DM and HC in peripapillary vessel density. This effect is more evident on peripapillary than macular superficial vascular density.

Peripapillary vessel density provides a greater diagnostic ability than macular vessel density in glaucoma patients, and in addition to being more clinically relevant in glaucoma, it may be more affected by these factors than macular vascular density, which should be considered in long-term studies in glaucoma patients.

## Figures and Tables

**Table 1:** Demographic, clinical, and ocular characteristics.

	Normal + Glaucoma Subjects (n = 317)	Normal (n = 162)	Glaucoma (n = 155)	*p* Value
Gender (M/F)	118/199	59/140	94/119	0.002 &
Age (yr)	58.27 ± 20.55	50.37 ± 21.17	66.53 ± 16.24	<0.0001 #
Patients taking glaucoma medications	52 (32.2%)	-	52 (32.2%)	-
Number of glaucoma medications	0.56 [0; 1]	-	1 [0; 2]	-
Glaucoma surgery	18 (11.6%)	-	18 (11.6%)	-
Mean Defect (dB)	2 [−0.25; 3.5]	0.7 [−2.05; 3.1]	2.5 [0.4; 5.2]	0.038 *
Arterial hypertension	123/194	34 (21%)	89 (57.4%)	<0.0001 &
Diabetes mellitus	44/273	14 (8.6%)	30 (19.3%)	0.005 &
Hypercholesterolaemia	90/127	20 (12.3%)	70 (45.2%)	<0.0001 &
Pseudophakia	88/229	34 (21%)	54 (34.8%)	0.004 &
Visual Acuity	0.79 ± 0.24	0.79 ± 0.27	0.74 ± 0.26	0.068 #
Sphere	0.34 ± 1.94	0.1 ± 1.96	0.60 ± 1.89	0.021 #
Astigmatism	−0.75 [−1.25; −0.5]	−0.75 [−1.25; −0.5]	−1 [−1.5; −0.5]	0.001 *
IOP GAT (mmHg)	16.62 ± 3.82	15.52 ± 2.79	17.77 ± 4.38	<0.0001 #
IOP iCare (mmHg)	16.79 ± 4.06	15.88 ± 3.05	17.73 ± 4.73	<0.0001 #
CCT (µ)	529.23 ± 65.79	532.33 ± 56.75	526.00 ± 74.13	0.393 #
SD-OCT parameters				
Disc area (mm^2^)	1.84 ± 0.30	1.85 ± 0.37	1.84 ± 0.38	0.937 #
Rim area (mm^2^)	1.21 ± 0.30	1.34 ± 0.24	1.08 ± 0.30	<0.0001 #
C/D	0.54 ± 0.19	0.47 ± 0.20	0.60 ± 0.17	<0.0001 #
Vertical C/D	0.51 ± 0.189	0.44 ± 0.17	0.58 ± 0.18	<0.0001 #
Cup Volume	0.21 ± 0.19	0.14 ± 0.14	0.27 ± 0.22	<0.0001 #
RNFL (µ)	89.57 ± 13.04	94.431 ± 9.30	84.48 ± 14.42	<0.0001 #
GCIPL (µ)	261.49 ± 30.17	258.25 ± 30.49	264.88 ± 29.55	0.050 #

& chi-square test; # Student’s *t*-test; * Median test; All above measurements are represented by mean ± SD except mean defect, which is expressed by median and [P25; P75]; GAT—Goldmann aplanation tonometry; IOP—Intraocular pressure; C/D—(cup to disc ratio); GCIPL = Average ganglion cell layer and inner plexiform layer; RNFL = Average retinal nerve fiber layer.

**Table 2:** Comparison of peripapillary and macular vessel density evaluated by OCTA between glaucoma and normal groups.

	Normal (n = 162)	Glaucoma (n = 155)	*p* Value
**Peripapillary OCTA Vessel Density**			
Whole Peripapillary Perfusion Density (%)	44.92 ± 1.65	43.22 ± 2.64	<0.0001
Whole Peripapillary Blood Flux Index	0.44 ± 0.03	0.41 ± 0.04	<0.0001
Peripapillary Perfusion Density in the superior quadrant (%)	43.22 ± 2.33	41.12 ± 3.64	<0.0001
Peripapillary Blood Flux Index in the superior quadrant	0.43 ± 0.03	0.40 ± 0.04	<0.0001
Peripapillary Perfusion Density in the inferior quadrant (%)	45.25 ± 2.28	41.90 ± 4.07	<0.0001
Peripapillary Blood Flux Index in the inferior quadrant	0.44 ± 0.03	0.40 ± 0.04	<0.0001
Peripapillary Perfusion Density in the temporal quadrant (%)	47.33 ± 2.82	46.61 ± 2.72	0.021
Peripapillary Blood Flux Index in the temporal quadrant	0.46 ± 0.42	0.42 ± 0.05	<0.0001
Peripapillary Perfusion Density in the nasal quadrant (%)	43.82 ± 2.33	42.85 ± 2.85	0.001
Peripapillary Blood Flux Index in the nasal quadrant	0.45 ± 0.04	0.41 ± 0.04	<0.0001
**Macular OCTA Vessel Density**			
Whole Macular Perfusion Density (%)	43.72 ± 4.55	40.88 ± 5.75	<0.0001
Macular Perfusion Density in the outer circle (%)	45.04 ± 4.71	41.94 ± 5.74	<0.0001
Macular Perfusion Density in the inner circle (%)	42.28 ± 4.81	39.96 ± 6.74	<0.0001
Macular Perfusion Density in the central circle (%)	21.38 ± 7.44	20.18 ± 8.35	0.177
Foveal Avascular Zone Area (mm^2^)	0.23 ± 0.11	0.21 ± 0.13	0.191
Foveal Avascular Perimeter (mm)	1.96 ± 0.50	1.96 ± 0.75	0.929
Acircularity Index	0.71 ± 0.09	0.68 ± 0.11	0.005

t-Student test; All above measurements are represented by mean ± SD.

**Table 3:** Correlation between peripapillary and macular vessel density indices analyzed with OCTA and demographic and clinical data and SD-OCT parameters.

	Whole Peripapillary Perfusion Density (%)	Peripapillary Perfusion Density in the Superior Quadrant (%)	Peripapillary Perfusion Density in the Inferior Quadrant (%)	Peripapillary Perfusion Density in the Temporal Quadrant (%)	Peripapillary Perfusion Density in the Nasal Quadrant (%)	Whole Macular Perfusion Density (%)	Macular Perfusion Density in the Outer Circle (%)	Macular Perfusion Density in the Inner Circle (%)	Macular Perfusion Density in the Central Circle (%)
Age (yr)	−0.397 (<0.0001) §	−0.305 (<0.0001) §	−0.397 (<0.0001) §	−0.221 (<0.0001) §	−0.171 (0.002) §	−0.478 (<0.0001) §	−0.484 (<0.0001) §	−0.388 (<0.0001) §	−0.254 (<0.0001) §
Glaucoma medications	−0.363 (<0.0001) *	−0.320 (<0.0001) *	−0.443 (<0.0001) *	−0.127 (0.024) *	−0.183 (0.001) *	−0.267 (<0.0001) *	−0.319 (<0.0001) *		
Mean Defect (dB)						−0.630 (0.002) *	−0.686 (<0.0001) *		
Visual Acuity		0.136 (0.016) §	0.171 (0.002) §			0.270 (<0.0001) §	0.277 (<0.0001) §	0.202 (<0.0001) §	0.196 (0.001) §
Sphere								−0.145 (0.010) §	−0.218 (<0.0001) §
Astigmatism	0.159 (0.009) *	0.195 (0.032) *	0.191 (0.002) *		−0.120 (0.048) *	0.336 (<0.0001) *	0.338 (<0.0001) *	0.218 (<0.0001) *	0.131 (0.020) *
IOP GAT (mmHg)							−0.142 (0.011) §		−0.142 (0.011) §
IOP iCare (mmHg)									
CCT (µ)	0.267 (<0.0001) §	0.269 (<0.0001) §	0.161 (0.004) §	0.134 (0.017) §	0.232 (<0.0001) §				
SD-OCT parameters									
Disc area (mm^2^)									
Rim area (mm^2^)	0.471 (<0.0001) §	0.351 (<0.0001) §	0.502 (<0.0001) §	0.259 (<0.0001) §	0.294 (<0.0001) §	0.204 (<0.0001) §	0.253 (<0.0001) §		−0.145 (0.010) §
C/D	−0.248 (<0.0001) §	−0.212 (<0.0001) §	−0.290 (<0.0001) §		−0.143 (0.011) §	−0.124 (0.028) §	−0.146 (0.009) §		
Vertical C/D	−0.327 (<0.0001) §	−0.249 (<0.0001) §	−0.371 (<0.0001) §	−0.181 (0.001) §	−0.174 (0.002) §	−0.165 (0.003) §	−0.202 (<0.0001) §		
Cup Volume	−0.276 (<0.0001) §	−0.220 (<0.0001) §	−0.361 (<0.0001) §		−0.129 (0.022) §		−0.133 (0.018) §		
RNFL (µ)	0.356 (<0.0001) §	0.429 (<0.0001) §	0.313 (<0.0001) §		0.253 (<0.0001) §	0.172 (0.002) §	0.204 (<0.0001) §		
GCIPL (µ)	−0.196 (<0.0001) §	−0.153 (0.006) §	−0.223 (<0.0001) §	0.136 (0.016) §	−0.157 (0.005) §	−0.115 (0.041) §	−0.137 (0.014) §		0.282 (<0.0001) §

§ Pearson Correlation Coefficient. * Spearman Correlation Coefficient. GAT—Goldmann aplanation tonometry. IOP—Intraocular pressure. C/D—(cup to disc ratio). GCIPL = Average ganglion cell layer and inner plexiform layer. RNFL = Average retinal nerve fiber layer.

**Table 4:** Linear regression analysis to evaluate the effect of glaucoma, SAH, HC and DM diagnosis and the most relevant demographic and clinical data on sPVD and sMVD.

	β	95%CI	*p*
**Whole Peripapillary Perfusion Density (%)**			
Diabetes mellitus	0.925	(0.293; 1.558)	0.004
Gender	1.190	(0.750; 1.631)	<0.0001
Pseudophakia	1.795	(1.311; 2.280)	<0.0001
Glaucoma	1.228	(0.798; 1.659)	<0.0001
**Peripapillary Perfusion Density in the superior quadrant (%)**			
Pseudophakia	2.082	(1.379; 2.784)	<0.0001
Gender	1.497	(0.857; 2.136)	<0.0001
Diabetes mellitus	1.153	(0.235; 2.070)	0.014
Glaucoma	1.542	(0.917; 2.166)	<0.0001
**Peripapillary Perfusion Density in the inferior quadrant (%)**			
Gender	1.886	(1.215; 2.558)	<0.0001
Pseudophakia	2.225	(1.376; 3.074)	<0.001
Glaucoma	2.530	(1.823; 3.238)	<0.0001
**Peripapillary Perfusion Density in the temporal quadrant (%)**			
Pseudophakia	0.942	(0.156; 1.727)	0.019
Age	−0.019	(−0.036; −0.002)	0.029
**Peripapillary Perfusion Density in the nasal quadrant (%)**			
Diabetes mellitus	1.205	(0.389; 2.020)	0.004
Gender	1.006	(0.438; 1.574)	0.001
Pseudophakia	1.151	(0.527; 1.776)	<0.0001
Glaucoma	0.576	(0.438; 1.574)	0.042
**Whole Macular Perfusion Density (%)**			
Age	−0.081	(−0.115; −0.047)	<0.0001
Hypercholesterolaemia	1.324	(0.015; 2.634)	0.047
**Macular Perfusion Density in the outer circle (%)**			
Age	−0.072	(−0.106; −0.039)	<0.001
Pseudophakia	1.921	(0.568; 3.274)	0.006
Arterial Hypertension	1.513	(0.216; 2.810)	0.022
Hypercholesterolaemia	1.549	(0.240; 2.858)	0.021
**Macular Perfusion Density in the inner circle (%)**			
Age	−0.112	(−0.142; −0.083)	<0.0001
**Macular Perfusion Density in the central circle (%)**			
Age	−0.125	(−0.173; −0.077)	<0.001
Pseudophakia	−2.384	(−4.595; −0.174)	0.035
**Foveal Avascular Zone Area (mm^2^)**			
Gender	0.044	(0.018; 0.070)	0.001
Arterial hypertension	0.048	(0.022; 0.074)	<0.001
**Foveal Avascular Perimeter (mm)**			
Arterial hypertension	0.192	(0.049; 0.336)	0.009
Gender	0.151	(0.007; 0.296)	0.040
**Acircularity Index**			
Age	−0.002	(−0.002; −0.001)	<0.0001

Variables entered in regression model: glaucoma, age, gender, pseudophakia, arterial hypertension, diabetes mellitus, hypercholesterolaemia. The variable included in β slope is the variable included in the backward step model. β = Beta slope; 95%CI = 95% confidence interval.

**Table 5:** Areas under receiver operating characteristic curves (AUROCs) for the variables measured using OCTA.

					Sensitivity at 95% Specificity						Sensitivity at 80% Specificity					
	AUROC	95%CI		*p*-Valor	Cut-Off	Sensitivity	95%CI		LR+	LR−	Cut-Off	Sensitivity	95%CI		LR+	LR−
RNFL (µ)	0.692	0.62	0.76	<0.001	80.0	0.43	0.33	0.53	8.7	0.59	86.0	0.53	0.43	0.63	2.66	0.58
Symmetry	0.738	0.67	0.8	<0.001	58.5	0.28	0.18	0.43	5.63	0.76	76.5	0.61	0.47	0.7	3.06	0.49
Rim area (mm^2^)	0.746	0.68	0.81	<0.001	0.98	0.44	0.33	0.59	8.81	0.59	1.14	0.61	0.5	0.72	3.03	0.49
Cup Volume	0.687	0.62	0.76	<0.001	0.5	0.16	0.08	0.39	3.12	0.89	0.27	0.44	0.32	0.61	2.21	0.7
Vertical C/D	0.716	0.65	0.78	<0.001	0.65	0.31	0.15	0.47	6.25	0.72	0.59	0.55	0.41	0.67	2.74	0.56
C/D	0.716	0.65	0.78	<0.001	0.48	0.27	0.13	0.47	5.32	0.77	0.41	0.54	0.43	0.65	2.73	0.57
Whole Peripapillary Perfusion Density (%)	0.708	0.64	0.78	<0.001	42.05	0.25	0.1	0.38	4.95	0.79	43.6	0.53	0.34	0.65	2.65	0.59
Whole Peripapillary Blood Flux Index	0.789	0.73	0.85	<0.001	0.39	0.29	0.18	0.41	5.85	0.74	0.42	0.65	0.46	0.77	3.26	0.43
Peripapillary Perfusion Density in the superior quadrant (%)	0.709	0.64	0.78	<0.001	38.95	0.19	0.09	0.39	3.89	0.85	41.6	0.46	0.32	0.63	2.32	0.67
Peripapillary Blood Flux Index in the superior quadrant	0.772	0.71	0.84	<0.001	0.38	0.31	0.15	0.48	6.27	0.72	0.41	0.64	0.48	0.78	3.19	0.45
Peripapillary Perfusion Density in the inferior quadrant (%)	0.788	0.73	0.85	<0.001	41.45	0.37	0.24	0.51	7.34	0.67	43.35	0.6	0.49	0.72	3.01	0.5
Peripapillary Blood Flux Index in the inferior quadrant	0.763	0.70	0.83	<0.001	0.39	0.27	0.17	0.4	5.36	0.77	0.42	0.56	0.39	0.69	2.8	0.55
Peripapillary Perfusion Density in the temporal quadrant (%)	0.558	0.48	0.64	0.146	42.15	0.05	0.01	0.14	1.1	0.99	45.05	0.29	0.14	0.39	1.47	0.88
Peripapillary Blood Flux Index in the temporal quadrant	0.769	0.7	0.83	<0.001	0.39	0.22	0.13	0.41	4.45	0.82	0.43	0.56	0.41	0.77	2.79	0.55
Peripapillary Perfusion Density in the nasal quadrant (%)	0.549	0.47	0.63	0.214	39.9	0.12	0.05	0.19	2.39	0.93	41.5	0.28	0.17	0.39	1.38	0.91
Peripapillary Blood Flux Index in the nasal quadrant	0.792	0.73	0.85	<0.001	0.39	0.27	0.14	0.5	5.47	0.76	0.42	0.59	0.46	0.73	2.95	0.51
Whole Macular Perfusion Density (%)	0.738	0.67	0.81	<0.001	35.6	0.12	0.06	0.3	2.39	0.93	33.6	0.52	0.32	0.69	2.61	0.6
Macular Perfusion Density in the outer circle (%)	0.751	0.69	0.82	<0.001	36.4	0.13	0.06	0.3	2.57	0.92	44.4	0.56	0.36	0.75	2.78	0.55
Macular Perfusion Density in the inner circle (%)	0.573	0.5	0.65	0.065	10.95	0.13	0.03	0.24	2.57	0.92	17.15	0.32	0.21	0.43	1.61	0.85
Macular Perfusion Density in the central circle (%)	0.629	0.55	0.71	0.001	32.7	0.13	0.06	0.36	2.57	0.92	41.25	0.47	0.33	0.59	2.33	0.67
Acircularity Index	0.552	0.47	0.63	0.192	0.54	0.10	0.03	0.26	2.02	0.95	0.65	0.29	0.2	0.4	1.43	0.89

AUROC, area under the ROC curve; LR, likelihood ratio.

## Data Availability

No new data were created. Archived Datasets available under requirements.

## Supplementary Materials

The following supporting information can be downloaded at: https://www.mdpi.com/article/10.3390/jcm12052071/s1, Comparison of 115 OAG patients and 115 age-matched healthy subjects. Table S1. Demographic, clinical, and ocular characteristics of 115 OAG patients and 115 age-matched healthy subjects. Table S2. Comparison of peripapillary and macular vessel density evaluated by OCTA between glaucoma and normal groups of 115 OAG patients and 115 age-matched healthy subjects. Table S3. Correlation between peripapillary and macular vessel density indices analyzed with OCTA and demographic and clinical data and SD-OCT parameters of 115 OAG patients and 115 age-matched healthy subjects. Table S4. Linear regression analysis to evaluate the effect of glaucoma, SAH, HC and DM diagnosis and the most relevant demographic and clinical data on sPVD and sMVD of 115 OAG patients and 115 age-matched healthy subjects.

## Abbreviations

AI	Acircularity index
AMD	Age-related macular degeneration
AUROCS	Areas under the ROC curves
BCVA	Best-corrected visual acuity
DM	Diabetes mellitus
FAZ	Foveal avascular zone
GCIPL	Ganglion cell layer and inner plexiform layer
GAT	Goldmann applanation tonometer
HC	Hypercholesterolemia
IOP	Intraocular pressure
sMVD	Macular vessel density on superficial vascular plexus
MD	Mean defect
ONH	Optic nerve head
OCT	Optical coherence tomography
OCTA	Optical coherence tomography angiography
sPVD	Peripapillary vessel density on superficial vascular plexus
POAG	Primary open-angle glaucoma
ROC	Receiver operating characteristics
RNFL	Retinal nerve fiber layer
SD	Standard deviation
SD-OCT	Spectral domain optical coherence tomography
SAH	Systemic arterial hypertension
