# Supplementary material for: Effect of Hypercholesterolemia, Systemic Arterial Hypertension and Diabetes Mellitus on Peripapillary and Macular Vessel Density on Superficial Vascular Plexus in Glaucoma
Source: J Clin Med. 2023 Mar 6;12(5):2071. doi: 10.3390/jcm12052071 (PMC10004387; doi:10.3390/jcm12052071)
Supplement: Supplementary file 1 [file jcm-12-02071-s001.zip › jcm-2235350-supplementary.pdf]

Table S1. Demographic, clinical, and ocular characteristics of 115 OAG patients and 115 age-matched healthy subjects.

	Normal + glaucoma subjects (n=230)	Normal (n=115)	Glaucoma (n=115)	P value
**Gender (M/F)**	92/138	40/75	52/63	0.139&
**Age (yr)**	62.59±13.48	61.40±14.10	63.40±12.78	0.182#
**Patients under glaucoma medications**	85 (37%)	-	85 (37%)	-
**Number of glaucoma medications**	1 [0;2]	-	1 [0;2]	-
**Glaucoma surgery**	11 (9.56%)	-	11 (9.56%)	-
**Mean Defect (dB)**	1 [-2;3]	-2 [-2;6]	1 [-1.25;-0.5]	0.542*
**Arterial hypertension**	98/132	34 (29.6%)	64 (55.6%)	<0.0001&
**Diabetes mellitus**	42/188	28 (24.3%)	14 (12.2%)	0.026&
**Hypercholesterolaemia**	68/162	20 (17.4%)	48 (41.7%)	<0.0001&
**Pseudophakia**	63/167	34 (29.6%)	29 (25.2%)	0.554&
**Visual Acuity**	0.76±0.24	0.74±0.26	0.78±0.22	0.230#
**Sphere**	0.59±1.90	0.48±1.82	0.70±1.98	0.021#
**Astigmatism**	-0.75 [-1.31;-0.5]	-0.75 [-1.5;-0.5]	-1 [-1.25;-0.5]	0.716*
**IOP GAT (mmHg)**	16.96±3.87	15.80±2.79	18.11±4.42	<0.0001#
**IOP iCare (mmHg)**	17.04±4.26	16.03±3.16	18.06±4.94	<0.0001#
**CCT (µ)**	531.38±55.04	529.27±62.67	533.49±46.34	0.562#
**SD-OCT parameters**				
**Disc area (mm^2)**	1.86±0.37	1.87±0.37	1.85±0.38	0.820#
**Rim area (mm2)**	1.21±0.31	1.32±0.25	1.09±0.32	<0.0001#
**C/D**	0.55±0.18	0.47±0.20	0.60±0.18	<0.0001#
**Vertical C/D**	0.53±0.18	0.49±0.17	0.58±0.19	<0.0001#
**Cup Volume**	0.22±0.20	0.15±0.15	0.28±0.23	<0.0001#
**RNFL (µ)**	89.41±13.11	94.76±9.60	85.06±14.66	<0.0001#
**GCIPL (µ)**	262.52±29.70	262.50±25.96	262.55±33.14	0.989#

&Chi-square test

# t-Student test

* Median test

All above measurements are represented by mean±SD except Mean Defect which are expressed by Median and [P25;P75]

GAT Goldmann aplanation tonometry

IOP Intraocular pressure

MD Mean defect; C/D (cup to disc ratio)

GCIPL= Average Ganglion cell layer and inner plexiform layer

RNFL= Average Retinal Nerve Fiber Layer

Table S2. Comparison of peripapillary and macular vessel density evaluated by OCTA between glaucoma and normal groups of 115 OAG patients and 115 age-matched healthy subjects.

	Normal (n=115)	Glaucoma (n=115)	P value
**Peripapillary OCTA Vessel Density**			
Whole Peripapillary Perfusion Density (%)	44.59±1.73	43.48±2.29	<0.0001
Whole Peripapillary Blood Flux Index	0.44±0.04	0.41±0.04	<0.0001
Peripapillary Perfusion Density in the superior quadrant (%)	42.89±2.44	41.45±3.18	<0.0001
Peripapillary Blood Flux Index in the superior quadrant	0.42±0.03	0.40±0.04	<0.0001
Peripapillary Perfusion Density in the inferior quadrant (%)	44.88±2.39	42.13±3.68	<0.0001
Peripapillary Blood Flux Index in the inferior quadrant	0.43±0.03	0.41±0.04	<0.0001
Peripapillary Perfusion Density in the temporal quadrant (%)	47.04±2.88	46.87±2.62	0.643
Peripapillary Blood Flux Index in the temporal quadrant	0.44±0.43	0.42±0.05	<0.0001
Peripapillary Perfusion Density in the nasal quadrant (%)	43.53±2.41	42.96±2.51	0.080
Peripapillary Blood Flux Index in the nasal quadrant	0.44±0.04	0.41±0.05	<0.0001
**Macular OCTA Vessel Density**			
Whole Macular Perfusion Density (%)	42.67±4.88	41.47±5.13	0.070
Macular Perfusion Density in the outer circle (%)	44.03±5.11	42.60±5.15	0.036
Macular Perfusion Density in the inner circle (%)	41.21±5.19	40.39±6.01	0.268
Macular Perfusion Density in the central circle (%)	20.08±7.48	19.99±7.81	0.929
Foveal Avascular Zone Area (mm2)	0.23±0.11	0.22±0.13	0.571
Foveal Avascular Perimeter (mm)	1.97±0.78	1.96±0.57	0.949
Acircularity Index	0.70±0.09	0.69±0.11	0.499

t-Student test

All above measurements are represented by mean ±SD

Table S3. Correlation between peripapillary and macular vessel density indices analyzed with OCTA and demographic and clinical data and SD-OCT parameters of 115 OAG patients and 115 age-matched healthy subjects.

	**Whole Peripapillary Perfusion Density (%)**	**Peripapillary Perfusion Density in the superior quadrant (%)**	**Peripapillary Perfusion Density in the inferior quadrant (%)**	**Peripapillary Perfusion Density in the temporal quadrant (%)**	**Peripapillary Perfusion Density in the nasal quadrant (%)**	**Whole Macular Perfusion Density (%)**	**Macular Perfusion Density in the outer circle (%)**	**Macular Perfusion Density in the inner circle (%)**	**Macular Perfusion Density in the central circle (%)**
**Age (yr)**	-0.232 (<0.0001)§	-0.193 (0.003)§	-0.299 (<0.0001)§			-0.401 (<0.0001)§	-0.410 (<0.0001)§	-0.300 (<0.0001)§	-0.192 (0.003)§
**Glaucoma medications**	-0.327 (<0.0001)*	-0.293 (<0.0001)*	-0.440 (<0.0001)*			-0.155 (0.019)*	-0.214 (<0.0001)*		
**Mean Defect (dB)**	-0.597 (0.024)*	-0.536 (0.048)*				-0.772 (0.001)*	-0.853 (<0.0001)*		
**Visual Acuity**					-0.208 (0.002)§	0.238 (<0.0001)§	0.242 (<0.0001)§	0.176 (0.007)§	0.193 (0.003)§
**Sphere**	0.135 (0.040)§			0.144 (0.029)§	0.176 (0.008)§			-0.145 (0.010)§	-0.241 (<0.0001)§
**Astigmatism**					-0.147 (0.026)*	0.229 (<0.0001)*	0.216 (0.001)*	0.211 (0.001)*	0.143 (0.031)*
**IOP GAT (mmHg)**									
**IOP iCare (mmHg)**					-0.131 (0.047)*				
**CCT (µ)**	0.151 (0.022)§	0.191 (0.004)§							
**SD-OCT parameters**									
**Disc area (mm2)**					0.136 (0.040)*				
**Rimarea (mm2)**	0.404 (<0.0001)§	0.253 (<0.0001)§	0.472 (<0.0001)§	0.190 (0.004)§	0.263 (<0.0001)§	0.130 (0.049)§	0.182 (0.006)§		-0.194 (0.003)§
**C/D**	-0.212 (0.001)§		-0.292 (<0.0001)§		-0.143 (0.030)§				
**Vertical C/D**	-0.255 (<0.0001)§		-0.343 (<0.0001)§		-0.137 (0.037)§		-0.140 (0.034)§		
**Cup Volume**	-0.212 (0.001)§	-0.134 (0.042)§	-0.332 (<0.0001)§		-0.129 (0.022)§		-0.133 (0.018)§		
**RNFL (µ)**	0.572	0.385	0.642	0.354	0.324	0.172	0.379	0.146	

	(<0.0001)§	(<0.0001)§	(<0.0001)§	(<0.0001)§	(<0.0001)§	(0.002)§	(<0.0001)§	(0.027)§	
**GCIPL (μ)**									0.289 (<0.0001)§

§ Pearson Correlation Coefficient.

* Spearman Correlation Coefficient.

GAT Goldmann aplanation tonometry

IOP Intraocular pressure

MD Mean defect; C/D (cup to disc ratio)

GCIPL= Average Ganglion cell layer and inner plexiform layer

RNFL= Average Retinal Nerve Fiber Layer

Table S4. Linear regression analysis to evaluate the effect of glaucoma, SAH, HC and DM diagnosis and the most relevant demographic and clinical data on sPVD and sMVD of 115 OAG patients and 115 age-matched healthy subjects.

	**β**	**95% CI**	**p**
**Whole Peripapillary Perfusion Density (%)**			
Diabetes mellitus	1.153	(0.523;1.783)	<0.0001
Gender	1.023	(0.544;1.501)	<0.0001
Pseudophakia	1.435	(0.898;1.972)	<0.0001
Glaucoma	0.919	(0.447;1.390)	<0.0001
**Peripapillary Perfusion Density in the superior quadrant (%)**			
Pseudophakia	1.701	(0.914;2.488)	<0.0001
Gender	0.922	(0.222;1.623)	0.010
Diabetes mellitus	1.483	(0.560;2.406)	0.002
Glaucoma	1.235	(0.545;1.926)	0.001
**Peripapillary Perfusion Density in the inferior quadrant (%)**			
Gender	1.730	(0.987;2.472)	<0.0001
Pseudophakia	1.814	(0.891;2.737)	<0.0001
Glaucoma	2.548	(1.815;3.282)	<0.0001
Age	-0.041	(-0.072;-0.011)	0.009
**Peripapillary Perfusion Density in the temporal quadrant (%)**			
Pseudophakia	0.834	(0.040;1.629)	0.040
**Peripapillary Perfusion Density in the nasal quadrant (%)**			

Diabetes mellitus	1.491	(0.678;2.304)	<0.0001
Gender	0.888	(0.271;1.505)	0.005
Pseudophakia	1.253	(0.483;2.023)	0.002
**Whole Macular PerfusionDensity (%)**			
Age	-0.130	(-0.179;-0.081)	<0.0001
**Macular Perfusion Density in the outer circle (%)**			
Age	-0.132	(-0.182;-0.082)	<0.0001
Arterial Hypertension	1.702	(0.343;3.061)	0.014
**Macular Perfusion Density in the inner circle (%)**			
Age	-0.125	(-0.177;-0.073)	<0.0001
**Macular Perfusion Density in the central circle (%)**			
Age	-0.109	(-0.181;-0.036)	0.003
**Foveal Avascular Zone Area (mm2)**			
Gender	0.047	(0.015;0.070)	0.004
Arterial hypertension	0.042	(0.011;0.074)	0.009
**Foveal Avascular Perimeter (mm)**			
Arterial hypertension	0.173	(-0.005;0.351)	0.056
**Acircularity Index**			
Age	-0.002	(-0.003;-0.001)	<0.0001

Variables entered in regression model: Glaucoma, age, gender, pseudophakia, arterial hypertension, diabetes mellitus, hypercholesterolaemia.

The variable included in β slope is the variable included in the backward step model.

β= Beta slope; 95% CI=95% Confidence interval.
